# Editorial: Insights in human and medical genomics: 2022

**DOI:** 10.3389/fgene.2023.1287894

**Published:** 2023-09-25

**Authors:** Jared C. Roach, Maxim B. Freidin

**Affiliations:** ^1^ Institute for Systems Biology, Seattle, WA, United States; ^2^ Department of Biology, School of Biological and Behavioural Sciences, Queen Mary University of London, London, United Kingdom

**Keywords:** genomics, Hill’s Criteria, multiomics, systems biology, epistemology, biological plausibility, correlation and causation, translational science

Genomics has matured considerably over the last decades. Increasingly, genome-scale genetic data are accompanied by other omic data: metabolomic, proteomic, epigenetic or methylomic, single-cell and spatial, lipidomic, diverse organ or tissue type, exosomal, imaging, *in vivo* and model organism, electronic health record (EHR), wearable device, cognitive or intrinsic capacity, and other health and healthspan data. At the frontiers of genomics, we increasingly welcome research that integrates multiple data types. More importantly: the maturation of genomics is not merely expansion into more dimensions of multidimensional omics dataspace, but also application of increasing wisdom in analysis and interpretation (Weiskittel et al., 2021).

**FIGURE 1:**
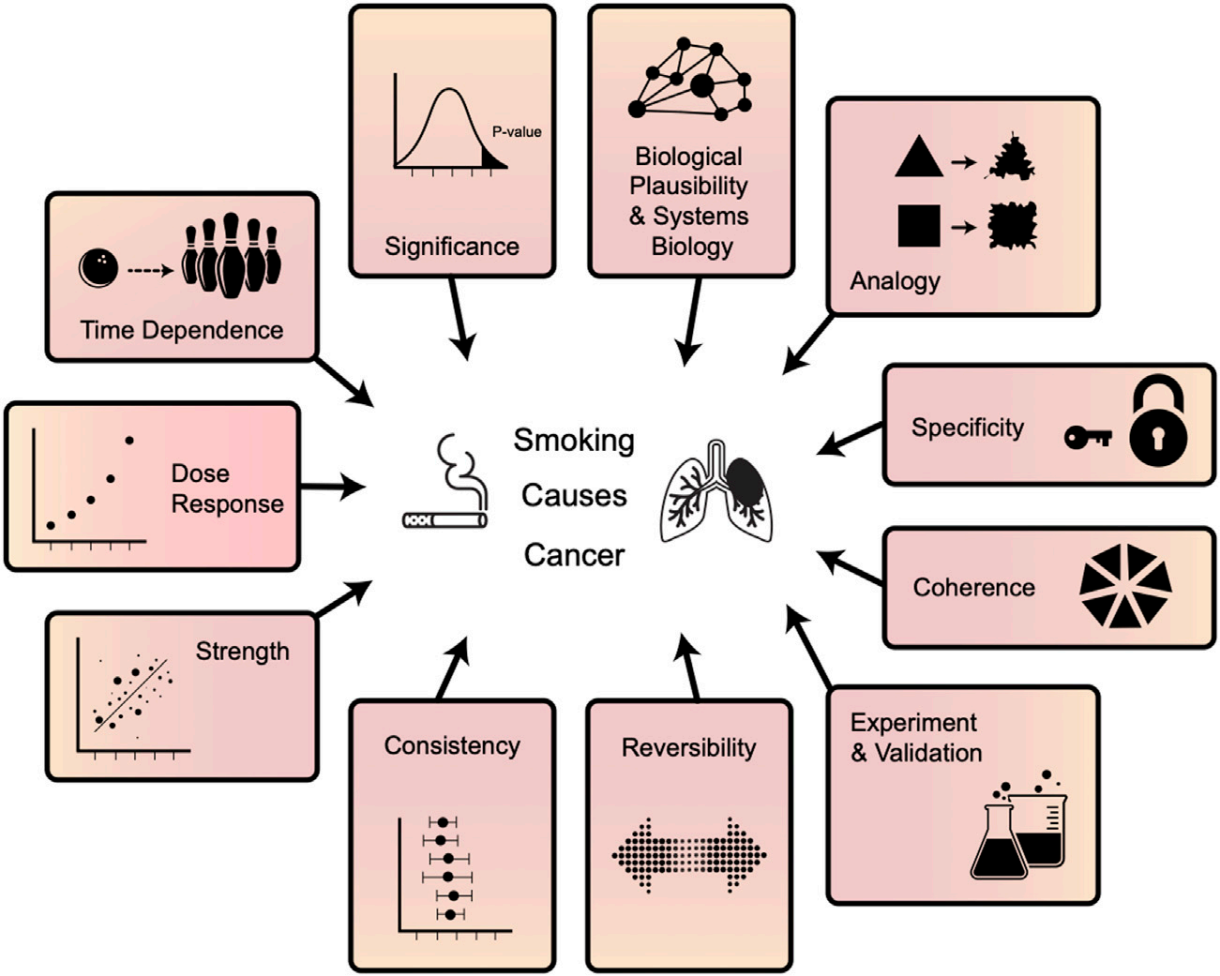
Multifaceted evidence will be necessary to best apply systems genomics to complex diseases. These lines of evidence were used in the 20th century to prove that smoking causes cancer and were termed “Hill’s Criteria”. The most important proofs of translational knowledge in this century will also require robust and diverse evidence. Both human and artificial intelligence (AI) analysis of basic research and translational medical and health data will leverage all of these pillars of knowledge.

Most modern translational science is built on two epistemological pillars: statistical significance and strength. However, the most impactful translational knowledge learned in the 20th century—that smoking causes cancer—necessitated the use of more diversified and robust epistemological techniques, collectively known as Hill’s criteria (Figure 1). Systems biology incorporates all of these lines of evidence, providing a holistic view of biomedical problems (Brigandt, 2013). These analyses particularly leverage the epistemological concepts of coherence and plausibility by fitting the ensemble of the data together consistently with prior knowledge. In the context of translational research, this emphasis is referred to as *biological plausibility* (Fedak et al., 2015). This synergy results from the integration of knowledge from various evidence streams, particularly multi-omics (Hood, 2013). Biological plausibility places current research coherently and consistently in the context of previous research. New observations are interpreted not only in the light of past and current *observations*, but also in the light of previous *knowledge*. Today, fuller implementation of epistemology for translational genomics is more feasible than it was a few years ago—because then we had much less *knowledge* of human molecular physiology.

For complex diseases, including many with substantial public health impacts such as dementia, allergies, infectious diseases, cancer, autoimmune disorders, and metabolic conditions, it is necessary to adapt the criterion of epistemological *coherence*. Hypotheses for complex diseases must assume multiple causes and potential interventions. Therefore, when seeking evidence of coherence, we should not anticipate a single unified coherent system but rather several potentially distinct coherent systems. Occam’s razor is rarely suitable for study of these complex diseases, as ample evidence already points to complex interactions shaped by non-parsimonious processes of evolution.

Understanding complex systems cannot be achieved by examining a single dimension at a time. Research portfolios centered on single-dimensional hypotheses would take centuries, incur exorbitant costs, and, in the case of clinical trials, necessitate more participants than could be recruited. In practice, embracing such a portfolio would mean no sufficiently complex disease could ever be adequately researched. Some variables, such as diet, offer an infinite range of possibilities, considering factors like doses, frequencies, and modalities. Synergy between variables may be important. Imagine being marooned on a desert island, and attempting to construct an escape raft using only individual materials at hand, such as driftwood, salvaged boards, coconuts, vines, and nails. Each of these materials, except for nails, may have some efficacy when used in isolation. However, even using the best material alone might not result in a successful raft, and the entire endeavor could be prematurely abandoned. The most effective raft is constructed by combining these materials in a multimodal manner, including nails—despite the fact that nails, in isolation, do not even float. Systems epistemology is required to build the raft, leveraging both past knowledge of how rafts can be constructed (consistency) and an understanding of how the available parts fit together in the current situation (coherency).

Correlation is not always causation, but sometimes is. Correlation of two variables (e.g., intervention and outcome) resulting from prospective randomization in an RCT is a robust argument for causation and has been considered a gold standard for clinical evidence (Ashcroft, 2004). Such a correlation must be mechanistic: the intervention *causes* the change in outcome. However, correlation from almost any other source of evidence introduces two additional possibilities. The outcome might cause the intervention or a third bioentity might cause both. Consequently, it is easy to dismiss correlations as epistemological evidence, often summarized with the phrase, “Correlation is not causation.” This statement can sometimes be taken too literally. *Correlation is evidence for causation* when it is combined with other evidence including dose-response, time-dependency, and biological-plausibility arguments (Hwang et al., 2005). Research that provides evidence for causation and mechanism leveraging multiple lines of epistemological inference will be increasingly valuable (Weith and Beyer, 2023). Although human analyses remain far from obsolete, we anticipate that “cyborg” analyses, which combine human insight with artificial intelligence (AI), will become increasingly compelling.

In this Research Topic, authors survey several aspects of these new frontiers in genomics. Varzari et al. leveraged classical techniques of family-based genetics to investigate the genetic influences on environmental exposure to mycobacterial infection and the resulting susceptibility to disease (Bennett et al., 2002). Savytska et al. contribute to tool development for studying the unexplored contribution of transposable elements to gene regulatory networks impacting complex traits and diseases (Kaplow et al., 2023). Fan et al. review the growing importance of omics in translational medicine by highlighting examples of non-coding RNAs as promising biomarkers (Skroblin and Mayr, 2014). Xulu et al. argue that multidimensional omics evidence is needed to best tailor personalized medicine for cancer, using evolution to anchor the *coherence* of these lines of evidence (Rivenbark et al., 2013). Diaz-Gonzalez et al. expand the spectrum of variants influencing Saethre-Chotzen syndrome (Paumard-Hernández et al., 2015). Shum et al. leverage high-throughput pairwise end-sequencing (Roach et al., 1995) and advances in bioinformatics to achieve newborn screening for spinal muscular atrophy (Nishio et al., 2023).

At *Frontiers in Genetics: Human and Medical Genomics* we continue to encourage submission of high-quality research. Priorities include encouraging authors from developing countries and disadvantaged authors from all countries. We solicit not just novel “first to the finish line” research, but also research that confirms previous findings, particularly in new and diverse populations. We look forward to crossing future genomics frontiers together.
